# Assessing early changes in plasma HER2 levels is useful for predicting therapeutic response in advanced breast cancer: A multicenter, prospective, noninterventional clinical study

**DOI:** 10.1002/cam4.5352

**Published:** 2022-10-24

**Authors:** Yi‐Kun Kang, Yi‐Ran Si, Jie Ju, Zhu‐Qing Jia, Nan‐Lin Hu, Hao Dong, Xue Wang, Jian Yue, Pei‐Di Jiang, Zhao‐Liang Li, Yun‐Yun Zhang, Yan Wang, Bing‐He Xu, Peng Yuan

**Affiliations:** ^1^ Department of VIP Medical Services, National Cancer Center/National Clinical Research Center for Cancer/Cancer Hospital Chinese Academy of Medical Sciences and Peking Union Medical College Beijing 100021 China; ^2^ Beijing Unicare Hospital Beijing China; ^3^ Questgenomics Nanjing China; ^4^ Gnomegen San Diego California USA

**Keywords:** breast cancer, digital PCR, plasma HER2, prediction threshold, therapy response

## Abstract

**Background:**

Early prediction of treatment response is crucial for the optimal treatment of advanced breast cancer. We aimed to explore whether monitoring early changes in plasma human epidermal growth factor receptor 2 (HER2) levels using digital PCR (dPCR) could predict the treatment response in advanced breast cancer.

**Methods:**

This was a multicenter, prospective, noninterventional clinical study of patients with advanced breast cancer. All enrolled patients underwent blood testing to measure the HER2 levels by digital PCR before treatment initiation and once every 3 weeks during the study. The primary endpoints were^a^ the diagnostic value of dPCR for detecting HER2 status in the blood and^b^ the relevance of potential changes in the plasma HER2 level at 3 weeks from baseline for predicting treatment response.

**Results:**

Overall, 85 patients were enrolled between October 9, 2018, and January 23, 2020. dPCR had a specificity of 91.67% (95% CI: 80.61% to 97.43%) for detecting HER2 amplification, and the area under the receiver operating characteristic (ROC) curve was 0.84 (*p* < 0.01). A clinically relevant specificity threshold of approximately 90%, which was equivalent to a ≥15% decrease in the plasma HER2 ratio at 3 weeks from baseline, showed a positive predictive value of 97.37% (95% CI: 77.11% to 98.65%) in terms of predicting clinical benefit. Patients whose plasma HER2 ratio was reduced by ≥15% had a longer median progression‐free survival (PFS) than those whose ratio was reduced by <15% (9.20 months vs. 4.50 months, *p* < 0.01).

**Conclusions:**

Early changes in the plasma HER2 ratio may predict the treatment response in patients with advanced breast cancer and could facilitate optimal treatment selection.

## Graphical abstract

Early prediction of treatment response is crucial for the optimal treatment of advanced breast cancer. This study found monitoring early changes in plasma human epidermal growth factor receptor 2 (HER2) levels using digital PCR (dPCR) could predict the treatment response in advanced breast cancer.

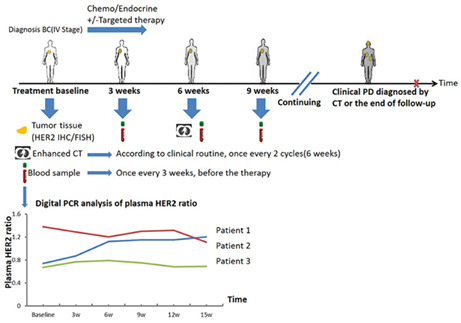

## 1 INTRODUCTION

Circulating tumor DNA (ctDNA) is currently the most widely used biomarker in liquid biopsy.^1^ As a minimally invasive detection method, liquid biopsy can quantitatively analyze the molecular changes in blood that are closely related to the occurrence and development of tumors. Human epidermal growth factor receptor 2 (HER2) is the most important gene marker for breast cancer, and HER2 amplification is an established risk factor for poor prognosis.^2^, ^3^ Research on circulating HER2 levels has generally focused on the clinical value of this parameter relative to that of pathological findings, which to date remain the gold standard for cancer diagnosis.^4^, ^5^ However, a more sensitive method than biopsy for detecting circulating HER2 amplification has yet to be established. Previous studies have shown that ctDNA levels are significantly influenced by the tumor burden^6^; thus, ctDNA levels fluctuate during treatment due to changes in tumor size. In this context, ctDNA levels are hypothesized to be an effective biomarker of treatment response.

Radiographic imaging remains the standard modality for evaluating treatment efficacy in solid tumors. However, the subjective impressions of physicians during imaging and differences in imaging equipment are limitations that cannot be excluded. Frequent exposure to imaging radiation is also discouraging to patients. Serum tumor markers (e.g., CEA, CA199, and CA153) have also been used as less invasive indicators for monitoring treatment efficacy. However, the long half‐life of these markers and lack of tumor specificity significantly limit their use in tumor evaluation.^7^, ^8^ Thus, other methods for the early prediction of treatment response are needed to identify patients who will optimally benefit from a specific treatment. Such methods will help in determining the suitability of changing the treatment regimen to improve outcomes or implementing maintenance treatment to lower toxicity. CtDNA has the advantages of a short half‐life and high tumor specificity. Such work can also be performed more frequently than radiographic evaluation. Therefore, ctDNA levels are a promising biomarker for monitoring treatment efficacy and even predicting the best treatment response. Previous studies have confirmed that the plasma HER2 extracellular domain levels in metastatic breast cancer patients reflected HER2 disease status.^9^, ^10^ This quantitative biomarker might help identifying patients without visceral disease profiting from a sequential treatment's modality. Recent studies also proved the changes in ctDNA from baseline can predict treatment response to neoadjuvant therapy in breast cancer.^11^ Furthermore, the dynamic changes in circulating HER2 also showed potential predictive value for evaluating treatment efficacy in gastric cancer.^12^ Therefore, the efficacy of dynamically monitoring plasma HER2 for predicting treatment response in advanced breast cancer is worthy of further research.

This study aimed to investigate the potential of early changes in plasma HER2 levels for predicting the best therapeutic response in advanced breast cancer. Ultimately, we aimed to obtain clinical evidence for establishing the dynamic monitoring of plasma HER2 as a feasible modality for predicting treatment response in breast cancer.

## 2 MATERIALS AND METHODS

### 2.1 Study design

This was a multicenter, prospective, observational clinical study that used dPCR to monitor the changes in plasma HER2 levels among advanced breast cancer patients from three cancer hospitals in Beijing, China. The study was approved by the Ethics Committee of the Cancer Hospital, Chinese Academy of Medical Sciences. The ethics registration number was NCC1824. This study was performed in accordance with the Declaration of Helsinki. Informed consent was obtained from all individual patients included in the study (NCT03947736).

### 2.2 Patients

We recruited patients with histologically confirmed stage IV breast cancer. The inclusion criteria were tissue histopathology of metastasis or recurrence within 1 month before enrollment, no treatment for metastases or recurrences, or documented disease progression from the most recent chemotherapy within 1 month before enrollment. The exclusion criteria were eligibility for surgery and ineligibility for or refusal of blood testing. The patient's pathology reports were reviewed to confirm the HER2 amplification status. HER2 positivity was defined, according to the American Society of Clinical Oncology/College of American Pathologists Clinical Practice Guideline Focused Update of HER2 testing in breast cancer, as either an immunohistochemistry (IHC) score of 3+ or 2+ with HER2 gene amplification detected using fluorescence in situ hybridization (FISH).^13^

### 2.3 Study protocol

The patients underwent blood testing at baseline before treatment initiation and then once every 3 weeks (usually at the end of week 3 or at beginning of week 4) until progressive disease (PD), the end of follow‐up, withdrawal of consent, or withdrawal by the investigator. Treatment was decided by the attending physician, and there were no restrictions on the treatment options. Treatment response was evaluated according to the Response Evaluation Criteria in Solid Tumors, version 1.1 (RECISTv1.1).^14^ Radiographic assessments of tumor response were conducted every two treatment cycles (6 weeks) until PD, end of follow‐up, or death.

### 2.4 Plasma preparation and ctDNA extraction

Peripheral blood (10 ml) was collected in PAXgene Blood ccfDNA tubes (Qiagen, Hilden, Germany). The collected whole blood was immediately centrifuged at 1900 × g for 15 min at room temperature (23°C to 27°C). The supernatant was collected and centrifuged again at 1900 × g for 10 min. The supernatant from the second centrifugation was considered the final plasma and was stored at −80°C until use. ctDNA was extracted from the plasma using a QIAamp Circulating Nucleic Acid Kit (Qiagen) according to the manufacturer's instructions.

### 2.5 dPCR analysis of plasma HER2

The HER2 amplification status was assessed according to the plasma HER2 ratio (plasma HER2 copy number to reference gene copy number) using a ProFlex2X Flat PCR system (Thermo Fisher Scientific) with the HER2 amplification detection kit Q02421 (Questgenomics).^15^ The information of reference gene was added to the Supplementary Methods. A total of 14.50 μl dPCR mixture was prepared with 5.80 μl ctDNA sample and RNase‐free water (approximately 5 ng ctDNA input), 7.25 μl dPCR Master Mix, and 1.45 μl HER2 amplification detection reaction solution. The dPCR mixture was loaded into chip wells using the Questgenomics Chip Loader, sealed, and then loaded onto the ProFlex2X Flat PCR system according to the manufacturer's instructions. The cycling conditions were as follows: 96°C for 10 min, 39 cycles of 60°C for 2 min and 98°C for 30 s, followed by a final extension step at 60°C for 2 min. The chip images were captured with the Questgenomics Biochip Reader and further analyzed using Cloud Software from Questgenomics.

### 2.6 Endpoints

There were two primary study endpoints: (a) the diagnostic value of dPCR for detecting HER2 amplification, which was evaluated with regard to the consistency in plasma HER2 levels between the findings of dPCR and tissue histopathology; and (b) the potential of the differences between the plasma HER2 ratio at 3 weeks from baseline for predicting treatment response from enrollment to PD, the end of follow‐up, withdrawal of consent, or withdrawal by the investigator. Additionally, this secondary endpoint evaluated the potential of the change in the plasma HER2 ratio from baseline for predicting PD earlier than computed tomography (CT).

### 2.7 Statistical analyses

The diagnostic value of dPCR for HER2 amplification was evaluated using the receiver operating characteristic (ROC) curve, sensitivity, specificity, and kappa coefficient. The Chi square or Fisher's exact test was used to assess the relationships between the plasma HER2 ratio and clinicopathological factors. The distributions of the change in the plasma HER2 ratio at 3 weeks among different response groups were plotted using Mann–Whitney *U*‐tests and Kruskal–Wallis tests. We identified the threshold for change at 3 weeks to achieve approximately 90% specificity. Then, the sensitivity, positive predictive value (PPV), and kappa coefficient associated with the threshold were compared using Fisher's exact test. Overall survival (OS) was defined as the interval between diagnosis and the last follow‐up or any‐cause death. Progression‐free survival (PFS) was defined as the interval between treatment initiation and PD, the last follow‐up (censored), or any‐cause death. Survival curves were plotted using the Kaplan–Meier method. Unadjusted and adjusted associations of the plasma HER2 ratio at baseline and a 15% decrease from baseline in the plasma HER2 ratio at 3 weeks with clinical outcomes were evaluated using the log‐rank test for clinical benefit (CB) and the Cox hazards regression models for PFS and OS. CB was defined as complete response (CR), partial response (PR), and stable disease (SD). As exploratory analysis, a paired t test was used to compare the median time for the plasma HER2 ratio to increase by 20% from baseline and the median time for PD to be detected on CT. All statistical analyses were performed using SPSS software (IBM SPSS version 22; IBM Corp., NY, USA) and GraphPad Prism (GraphPad Software Inc., San Diego, CA, USA, version 7.0). All tests were two‐sided, and *p* < 0.05 was considered statistically significant.

## 3 RESULTS

### 3.1 Patient characteristics

**FIGURE 1:**
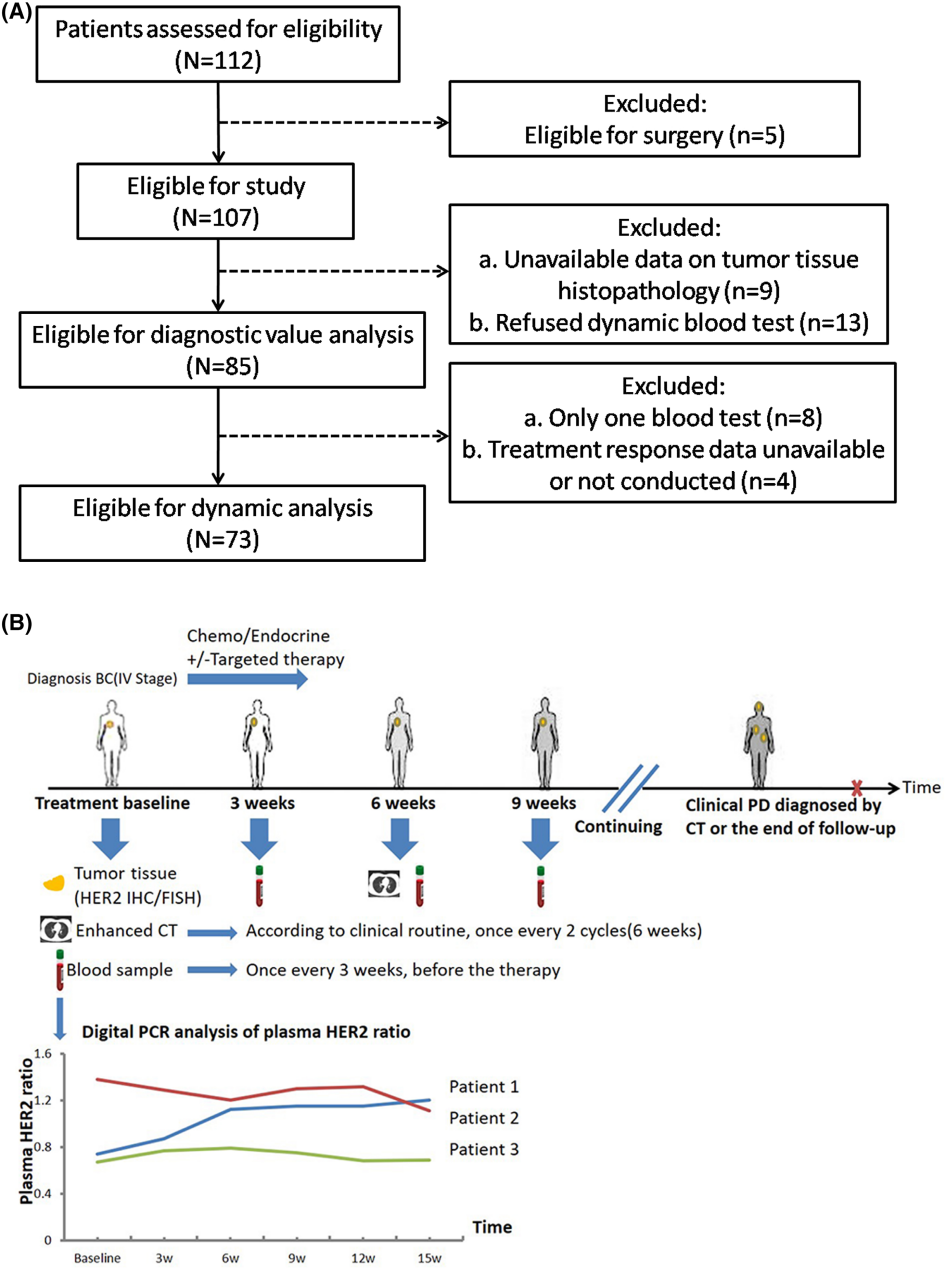
Patient inclusion flowchart (A) and sampling schedule (B).

A total of 85 patients with metastatic or recurrent breast cancer were enrolled between October 9, 2018, and January 23, 2020 (Figure 1A). The median follow‐up time was 8.5 months (range, 1.5–14.0 months). At the end of the study (January 23, 2020), 439 blood samples from 85 patients were collected. Twelve of these were excluded because they only completed the baseline blood sample collection (8 patients) or the data on treatment response were unavailable (4 patients). The remaining 73 patients underwent at least two blood sample collections and at least one treatment evaluation. At the end of the follow‐up, 39 (48.15%) patients developed PD, 42 (51.85%) were still undergoing treatment, and 4 patients were excluded due to the lack of treatment evaluation data. No patient in this study achieved CR. The baseline characteristics of 85 patients are presented in Table 1. All the patients were women, and the median age was 53 years old (range: 34–69 years old). There were 37 (43.53%) patients with tumor tissue HER2 amplification and 48 (56.47%) patients without amplification. Furthermore, 45 (52.94%) patients had ≥5 metastases and 36 (42.35%) patients were treated with chemotherapy/endocrine treatments combined with anti‐HER2 targeted therapy. All patients complied with the schedule for blood sample collection (Figure 1B).

**TABLE 1:** Clinical characteristics of the total enrolled patients (*N* = 85)

Characteristics	*N* (%)
Age (years), median (range)	53 (34–69)
Menopausal status	
Premenopausal	40 (47.06%)
Postmenopausal	45 (52.94%)
Degree of differentiation	
High and moderate	45 (52.94%)
Low	40 (47.06%)
N stage	
pN1‐N2	16 (18.82%)
pN3	69 (81.18%)
Hormone receptor status	
ER and/or PR positive	49 (57.65%)
ER and PR negative	36 (42.35%)
HER2 status of tumor tissue	
IHC 3+/2+ and FISH+	37 (43.53%)
IHC 0/1+/2+ and FISH‐	48 (56.47%)
Phenotype	
Triple‐positive	22 (25.88%)
HR‐, HER2+	15 (17.65%)
HR+, HER2‐	27 (31.76%)
Triple‐negative	21 (24.71%)
Ki‐67 index	
<14%	33 (38.82%)
≥14%	52(61.18%)
No. of metastasis sites	
<5	40 (47.06%)
≥5	45 (52.94%)
Previous treatment (before 1 month)	
Chemotherapy alone	30 (35.29%)
Chemotherapy+anti‐HER2	27 (31.76%)
Endocrine treatments+anti‐HER2	9 (10.59%)
Endocrine treatments+CDK4/6 inhibitors	15 (17.65%)
Others	4 (4.71%)
Previous lines of therapy in the metastatic setting	
0	10 (11.76%)
1	35 (41.18%)
2	29 (34.12%)
≥3	11 (12.94%)

### 3.2 Diagnostic value of dPCR for HER2 amplification in the blood

**FIGURE 2:**
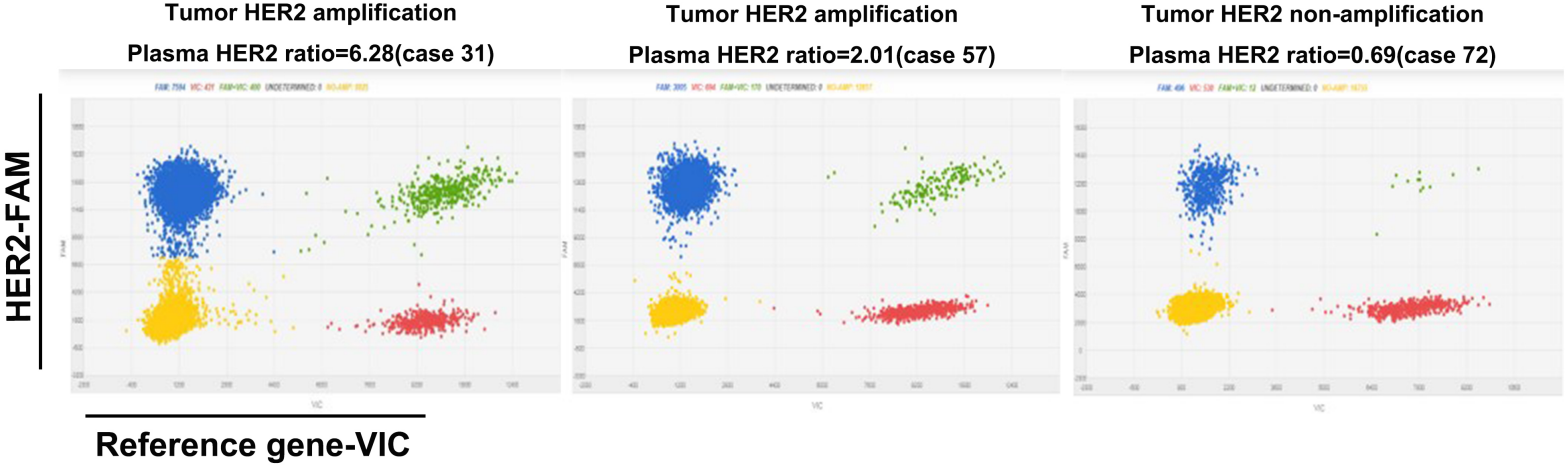
Scatter plot of dPCR detection of plasma HER2. HER2: Reference gene (plasma HER2 ratio) for detecting HER2 status using dPCR. Representative plots of the plasma HER2 ratio via dPCR analysis in patients with tumor HER2 amplification (left and middle panel) and non‐amplification (right panel). In each subfigure, the four quadrants represent droplets with HER2 DNA only (top left), droplets with both HER2 and reference gene DNA (top right), droplets with reference gene DNA only (bottom right), and droplets with no DNA (bottom left).

**FIGURE 3:**
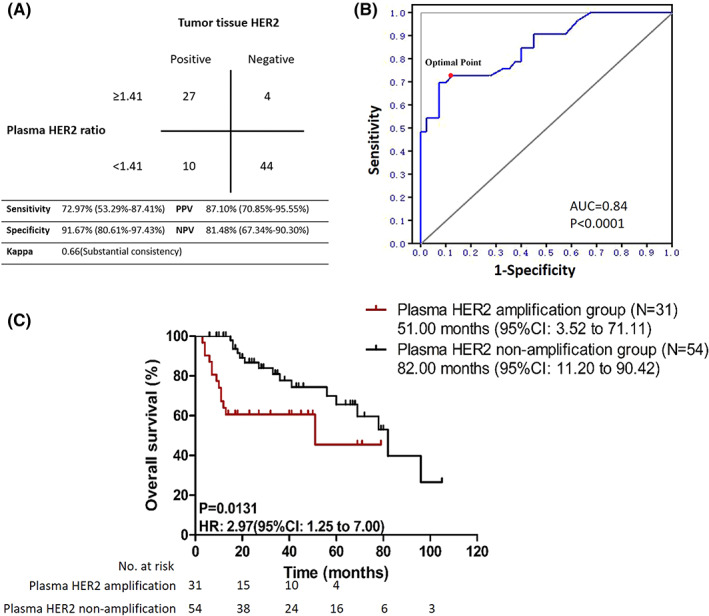
Diagnostic and prognostic value of HER2 amplification detected using dPCR. (A) ROC analysis with an AUC of 0.84 (*p* < 0.01). The optimal cutoff value was determined to be 1.41. (B) With a 1.41 cutoff value as the threshold, the plasma HER2 ratio of 85 patients in the cohort was analyzed (*p* < 0.01, Fisher's exact test). (C) Kaplan–Meier OS curves for patients with plasma HER2 ratios ≥1.41 and <1.41 at baseline (*p* = 0.01).

In this study, design, all 85 enrolled patients received a baseline dPCR analysis of HER2 amplification in ctDNA. The plasma HER2 ratio by dPCR was 6.24 ± 11.91 and 1.11 ± 0.28 in HER2 amplified group and HER2 nonamplified group, respectively (*p* < 0.05). The detection results of the plasma HER2 ratio by dPCR in three representative patients are shown in Figure 2. The results of ROC curve analysis showed that dPCR had a sensitivity of 72.97% (95% CI: 53.29% to 87.41%) for detecting HER2 amplification, with a specificity of 91.67% (95% CI: 80.61% to 97.43%), a kappa coefficient of 0.66 (Figure 3A), and an area under the ROC curve of 0.84 (*p* < 0.01; Figure 3B). We set the cutoff value in order to get the best Youden index, which was defined as “Sensitivity + Specificity – 1”. Therefore, the optimal cutoff value was determined to be 1.41. Accordingly, the patients were divided into a plasma HER2 amplification group (baseline plasma HER2 ratio ≥1.41) and a plasma HER2 non‐amplification group (baseline plasma HER2 ratio <1.41).

We then compared the clinicopathological features between the two groups (Table S1). Aside from the IHC and FISH parameters, other indicators of tumor burden were also significantly different between the two groups: primary lesion size (*p* < 0.01), long diameter of the target lesion (*p* < 0.01), and number of metastases (*p* < 0.01). With respect to prognosis, the plasma HER2 amplification group showed a worse OS than the plasma HER2 non‐amplification group, and the median OS was 51 months (95% CI: 3.52 to 71.11) vs. 82 months (95% CI: 11.20 to 90.42) (*p* < 0.01) (Figure 3C). The Cox prognostic model indicated that a plasma HER2 ratio of ≥1.41 was an adverse prognostic factor in patients with advanced breast cancer (HR = 1.90, 95% CI: 1.13 to 2.52, *p* < 0.01, shown in Table 2).

**TABLE 2:** Univariate and multivariate Cox regression analyses of the predictive factors of mortality risk for patients with metastatic or recurrent BC

Characteristic	Univariate HR (95% CI)	*p* value	Multivariate HR (95% CI)	*p* value
No. of metastasis	1.56(0.91–3.22)	0.11	1.53(1.14–2.57)	<0.01
Degree of differentiation	1.21 (0.90–1.62)	0.34		
PR	0.78 (0.45–1.59)	0.57		
ER	0.74(0.43–1.48)	0.23	0.72(0.51–0.92)	0.04
HER2 status on IHC/FISH	1.45 (0.87–1.53)	0.05	1.49(1.07–1.51)	<0.01
Ki‐67 index	0.73(0.50–1.12)	0.32		
Plasma HER2 ratio at baseline	1.88(0.99–2.87)	0.05	1.90(1.13–2.52)	0.04
With or without visceral metastasis	1.66(1.19–2.23)	0.03	1.72(1.20–2.13)	<0.01

### 3.3 Change in the plasma HER2 ratio at 3 weeks from baseline for predicting the best treatment response

**FIGURE 4:**
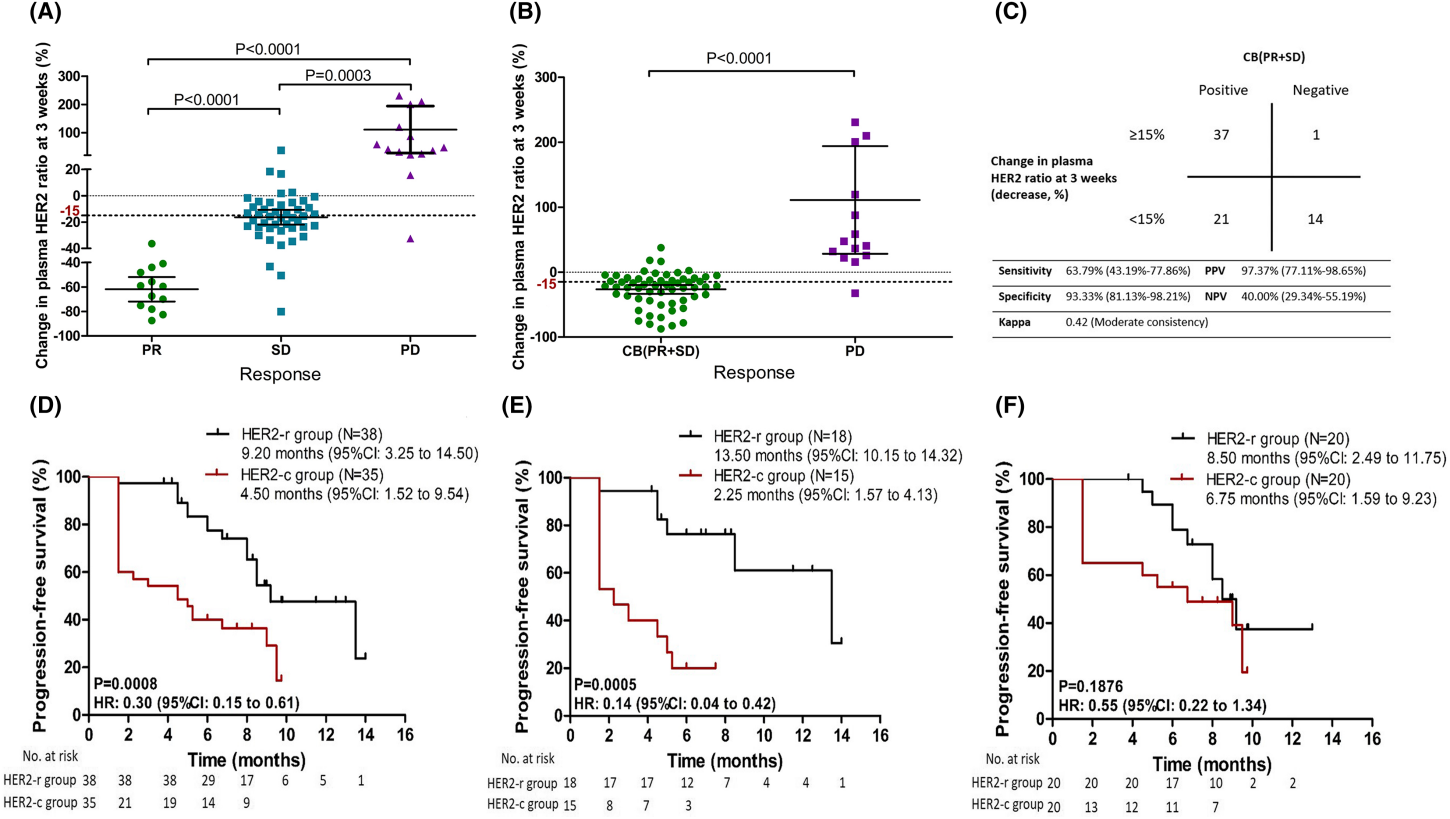
Changes in the plasma HER2 ratio at 3 weeks from baseline can predict the best treatment response. (A, B) Changes in the plasma HER2 ratio at 3 weeks from baseline are shown for patients grouped by treatment response. Each data point represents the percent change in the plasma HER2 ratio for a single patient. Horizontal bars represent the median, and error bars indicate the 95% CI. (C) With 15% as the threshold, using a ≥15% decrease in the plasma HER2 ratio at 3 weeks from baseline for predicting CB was analyzed (*p* < 0.01, Fisher's exact test). (D) Kaplan–Meier curves of PFS in the HER2‐r group and HER2‐c group (*p* < 0.01). (E) Kaplan–Meier curves of PFS in the HER2‐r group and HER2‐c group among those with tissue HER2 ratio amplification (*N* = 33, *p* = 0.03). (F) Kaplan–Meier curves of PFS in the HER2‐r group and HER2‐c group among those with tissue HER2 ratio non‐amplification (*N* = 40, *p* = 0.19).

A follow‐up study monitoring the change in the plasma HER2 ratio to predict therapy response was conducted in 73 patients who had at least one valid radiographic evaluation. The clinical characteristics of 73 patients are shown in Table S2. Based on the final data analysis, we found that the patients who achieved PR showed a more significant decrease in the plasma HER2 ratio at 3 weeks than patients who achieved SD (61.90 ± 4.57% vs. 16.35 ± 2.77%, *p* < 0.01) (Figure 4A). Meanwhile, patients with PD showed a significant increase in the plasma HER2 ratio (median: 111.10 ± 38.66%). The change in the plasma HER2 ratio also predicted CB (PR + SD). The decrease in the plasma HER2 ratio at 3 weeks was significantly different between patients with CB and PD (Figure 4B). A specificity threshold of approximately 90% equated to a decrease of ≥15% in the plasma HER2 ratio at 3 weeks from baseline detected using the dPCR method.^16^ Under this threshold, no more than 1 of every 10 patients who achieved CB from therapy would not be identified with the proposed dPCR method. A reduction of ≥15% in the plasma HER2 ratio at 3 weeks was defined as HER2‐reduced (HER2‐r). Meanwhile, a reduction by <15%, no change, or an increase was defined as HER2‐continued (HER2‐c). HER2‐r had a sensitivity of 63.79% (95% CI: 43.19% to 77.86%) for predicting CB. The PPV was 97.37% (95% CI: 77.11% to 98.65%), and the kappa coefficient was 0.42 (*p* < 0.01; Figure 4C). Furthermore, PFS was significantly better in HER2‐r patients than in HER2‐c patients (HR: 0.30, 95% CI: 0.15 to 0.61, *p* < 0.01); the median PFS in the HER2‐r group was 9.20 months (95% CI: 3.25 to 14.50), whereas it was only 4.50 months (95% CI: 1.52 to 9.54) in the HER2‐c group (Figure 4D). After adjusting for the treatment regimen and line of treatment, HER2‐r was found to be a significant factor associated with CB (OR = 4.14, 95% CI: 2.05 to 8.13; *p* < 0.01) and PFS (HR = 0.79, 95% CI: 0.51 to 0.92, *p* < 0.01) (Table 3). Next, we assessed the predictive value of the 15% threshold in patients with different baseline tissue HER2 amplification statuses. In the tissue HER2 amplification group, HER2‐r had a sensitivity of 73.91% (95% CI: 63.23% to 80.45%) for predicting CB, with a kappa coefficient of 0.56 (*p* = 0.02; Figure S1A,B). These results were similar to those in the general population. In the HER2 non‐amplification tissue group, the 15% threshold showed poor predictive performance for CB, with a kappa coefficient of only 0.25 (*p* = 0.03; Figure S1C,D). A comparison of the PFS between HER2‐r patients and HER2‐c patients showed that the PFS was better in the patients who achieved HER2‐r in the tissue HER2 amplification group (*p* < 0.01; Figure 4E). However, the *p* value for the HER2 non‐amplification group was not statistically significant (*p* = 0.19; Figure 4F). We also explored the difference between the plasma HER2 ratio at 6 weeks and baseline. At 6 weeks, the change in the plasma HER2 ratio was also correlated with CB (*p* < 0.01; Figure S1E,F). However, this study was initially designed to analyze the difference at 3 weeks as the primary endpoint.

**TABLE 3:** Unadjusted and adjusted associations between a 15% decrease in the plasma HER2 ratio at 3 weeks (HER2‐reduced/HER2‐r^a^) and clinical outcomes

Outcome	Unadjusted/univariate			Adjusted/multivariate^b^	
	OR/HR (95% CI)	*p* value		OR/HR (95% CI)	*p* value
PR	—^c^	—		—^c^	—
CB(PR + SD)	OR = 3.86(1.92–7.73)	<0.01		OR = 4.14(2.05–8.13)	<0.01
PFS	HR = 0.71(0.49–0.89)	<0.01		HR = 0.79(0.51–0.92)	<0.01

^a^ HER2‐r: 15% decrease in the plasma HER2 ratio at 3 weeks from baseline.

^b^ Adjusted based on the treatment and previous lines of chemotherapy in the metastatic setting.

^c^ Cannot be analyzed because all patients with PR had a ≥15% decrease in the plasma HER2 ratio at 3 weeks.

### 3.4 Plasma HER2 ratio for predicting PD earlier than CT

**FIGURE 5:**
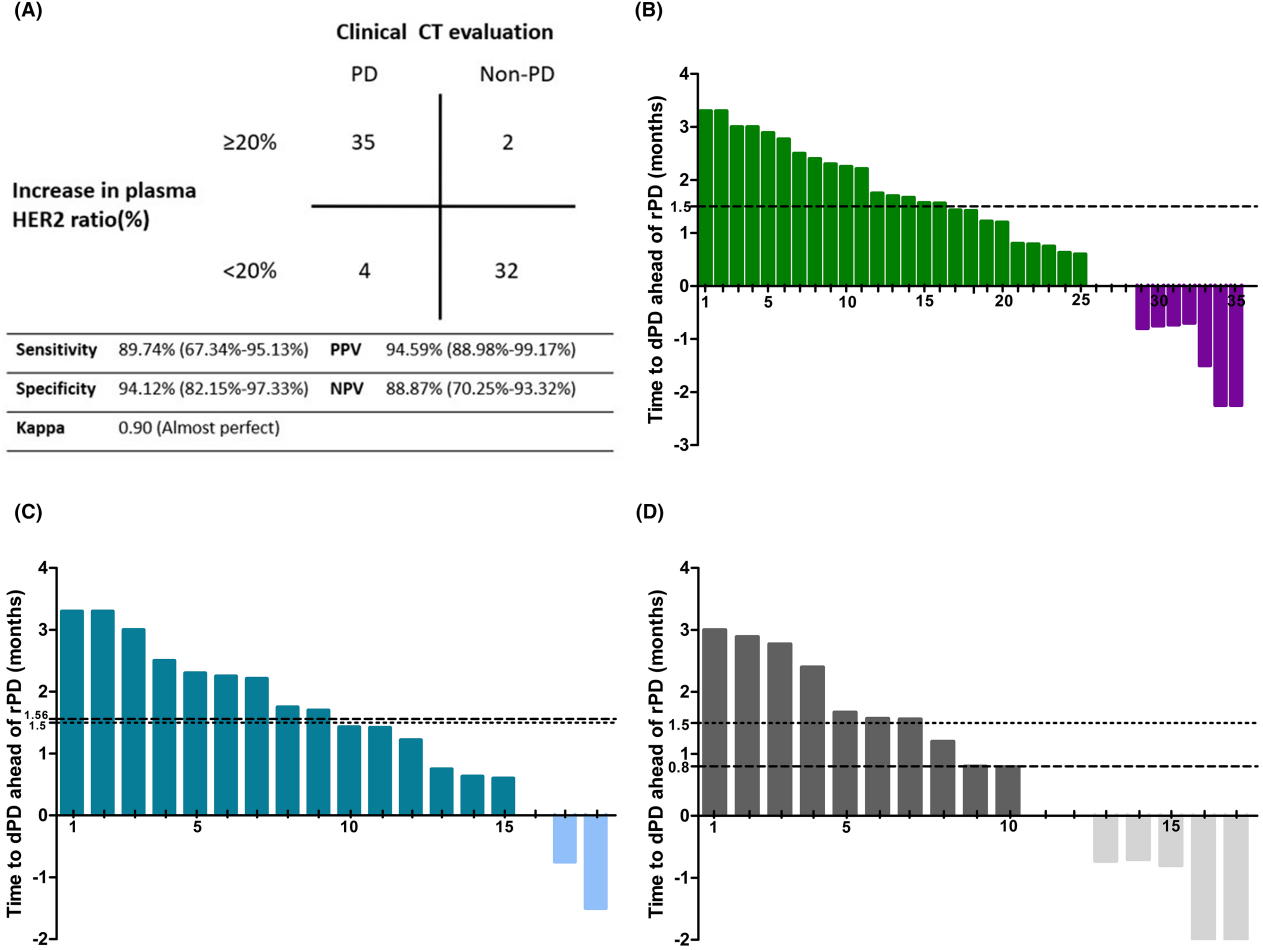
Plasma HER2 ratio can predict PD earlier than CT. (A) With 20% as the threshold, using a ≥20% increase in the plasma HER2 ratio at 3 weeks from baseline for predicting PD was analyzed (*p* < 0.01, Fisher's exact test). (B) Waterfall plots of time to dPD ahead of rPD in all the patients with rPD and whose plasma HER2 ratio increased by ≥20% from baseline (*N* = 35, *p* = 0.03). (C) Waterfall plots of time to dPD ahead of rPD in the patients with tissue HER2 ratio amplification (*N* = 14, *p* < 0.01). (D) Waterfall plots of time to dPD ahead of rPD in the patients with tissue HER2 ratio non‐amplification (*N* = 21, *p* < 0.01).

To investigate the early predictive value of the plasma HER2 ratio for radiographic PD (rPD), we defined a 20% increase in the plasma HER2 ratio as the detected PD (dPD). The threshold had a sensitivity of 89.74% (95% CI: 67.34% to 95.13%), a specificity of 94.12% (95% CI: 82.15% to 97.33%), and a PPV of 94.59% (95% CI: 88.98% to 99.17%) for predicting rPD. The kappa coefficient was 0.90 (*p* < 0.01) (Figure 5A). At the end of follow‐up, 35 patients with rPD showed a ≥20% increase in the plasma HER2 ratio from baseline. The median time from treatment initiation to rPD was 5.47 ± 0.36 months, and the median time to dPD was 3.97 ± 0.34 months. The median time for dPD ahead of rPD was 1.5 months (*p* = 0.03; Figure 5B). We further explored the predictive value of the 20% threshold in patients with different baseline tissue HER2 amplification statuses. The results indicated a higher predictive accuracy in the tissue HER2 amplification group than in the non‐amplification group (kappa coefficient: 0.94 vs. 0.62, Figure S2A,B). Furthermore, the median time difference of dPD ahead of rPD was greater in the plasma HER2 amplification group than in the non‐amplification group (1.56 ± 0.14 months vs. 0.80 ± 0.17 months; Figure 5C,D).

## 4 DISCUSSION

This study shows that HER2 has potential predictive value for evaluating treatment efficacy in advanced breast cancer. We discovered that dPCR is an effective diagnostic tool for measuring plasma HER2 levels and HER2‐r has potential value for predicting a positive treatment response in advanced breast cancer patients. HER2‐r showed a PPV of more than 97% for predicting CB, and all patients who achieved PR had a > 15% decrease in the plasma HER2 ratio at 3 weeks from baseline. Furthermore, only 1 of the 15 patients who did not achieve CB had a ≥ 15% decrease in the plasma HER2 ratio at 3 weeks. The clinical value of the HER2 ratio was further demonstrated by the longer PFS of patients with HER2‐r status relative to those with HER2‐c status.

Previous studies have confirmed that the plasma HER2 ratio determined via dPCR can be used to monitor the effects of treatments in patients with HER2‐positive gastric cancer.^17^, ^18^ Other studies have also explored the dynamic changes in ctDNA in the peripheral blood of breast cancer patients.^19^, ^20^ However, the results of these studies were limited to the finding that changes in ctDNA levels were related to treatment response, and the effective detection threshold was not determined.^21^, ^22^ Our findings indicate that monitoring the plasma HER2 ratio could offer early insights into patient response to treatment. In addition, the subgroup analysis in our study showed that HER2‐r had better sensitivity for predicting CB in the tissue HER2 amplification group than in the tissue HER2 non‐amplification group. More than half of the enrolled patients had HER2‐negative breast cancer by tissue in our study. The tissue IHC/FISH represents the current standard for HER2 assessment; thus, profiling the HER2 status in ctDNA in tissue‐HER2‐negative cases might be confusing. However, according to the findings presented here, the dynamic detection of changes in the plasma HER2 ratio can also be applied to tissue‐HER2‐negative patients to assess the treatment response due to changeable heterogeneity of HER2. However, in the HER2‐negative group, the sensitivity was worse than that of patients with HER2‐positive tissues.

Currently, the routine clinical review cycle for advanced breast cancer is once every 6 to 8 weeks. Our less‐invasive dPCR assay for the HER2 marker can be helpful for establishing a potential available threshold for the patient's therapy response within the first 3 weeks of treatment initiation. Early identification of patients who are not responding to therapy would enable an earlier change in the treatment course, increasing the possibility of patients benefiting from treatment and reducing toxicity from ineffective therapy. Therefore, the results of this study address the need for a potential threshold of the plasma HER2 ratio for predicting CB. Furthermore, our results provide an effective and less‐invasive monitoring method for the long‐term treatment of advanced breast cancer patients, especially in those with tissue HER2 amplification at baseline.

Our secondary endpoint of the usefulness of dPCR for predicting PD earlier than CT was also met. Patients whose plasma HER2 ratio increased by ≥20% from baseline had a 94.59% risk of achieving PD. The median time from treatment initiation to dPD was 1.5 months, which was significantly earlier than the median time to rPD. The median time of dPD ahead of rPD in the tissue HER2 amplification group was also earlier than that in the non‐amplification group. This result will further help clinicians determine the specific time by which patients will develop PD and provide physicians with the opportunity to change the treatment regimen to optimize treatment outcomes.

We also analyzed the consistency in plasma HER2 status between dPCR and histopathology. Despite the low sensitivity, dPCR showed 91.67% specificity for detecting HER2 levels in the blood, and the PPV was 87.10%. In this study, a total of 10 patients with HER2 positive status were scored with a low HER2 ratio by dPCR at baseline, which might result from that none of them had brain metastases and third‐line treatment was not applied. Therefore, these 10 patients might have relatively low tumor burden, leading to low level of ctDNA. Previous studies have confirmed that the proportion of circulating tumor cells and cell mutations are greatly influenced by the overall tumor burden.^23^, ^24^ Since the plasma HER2 ratio changes during the course of treatment, HER2 amplification in the peripheral blood might be affected by various factors across the treatment stages. It should be noted that attempting to completely replace tissue histopathology with dPCR is not ideal for detecting HER2 in the peripheral blood. Other highly sensitive indicators and detection methods are still needed.

Although this study was designed as a prospective trial, we actually conducted retrospective correlations between the HER2 ratio and therapy response, and the cutoff points were not predetermined. Thus, some limitations were inevitable in this study. First, this was an exploratory study with a small number of patients, and the findings should be verified in a separate validation dataset, which will require a large number of additional patients. We planned a next‐step study to improve the confidence in these findings. Second, approximately half of the patients enrolled in our study were HER2‐negative based on tissue, yet it seems that changes in the plasma HER2 ratio were still well correlated with the treatment response in our study and deserve significant further investigation. Third, we did not detect the HER2 level in normal heathy people as a control, which might be difficult to compare with other research directly. Fourth, due to the limited sample size, we did not include the lines of treatment and other tumor subtypes in the prognostic analysis. Fifth, due to the limited sample size, we failed to provide the lower limit for ctDNA from the tumor in a plasma sample. Finally, this study did not compare the differences between plasma levels of HER2 and plasma levels of other clinical tumor markers (e.g., CEA, CA153, and CA125).

In conclusion, we found that the changes in the plasma HER2 ratio at 3 weeks were indicative of the treatment response in advanced breast cancer patients. Furthermore, the change in the plasma HER2 ratio could also predict PD earlier than CT.

## AUTHOR CONTRIBUTIONS

**Yi‐Kun Kang:** Data curation (equal); formal analysis (equal); writing – original draft (equal). **Yiran Si:** Data curation (equal); formal analysis (equal). **Jie Ju:** Methodology (equal). **Zhu‐Qing Jia:** Funding acquisition (equal); investigation (equal). **Nan‐Lin Hu:** Methodology (equal). **Hao Dong:** Methodology (equal). **Xue Wang:** Investigation (equal). **Jian Yue:** Investigation (equal); methodology (equal). **Pei‐Di Jiang:** Data curation (equal). **Zhao‐Liang Li:** Data curation (equal). **Yun‐Yun Zhang:** Methodology (equal). **Yan Wang:** Investigation (equal). **BingHe Xu:** Funding acquisition (equal); writing – review and editing (equal). **Peng Yuan:** Conceptualization (equal); writing – review and editing (equal).

## FUNDING INFORMATION

This study was supported by the National Natural Science Foundation of China (81672634, 82172650), Beijing Medical Award Foundation (YXJL‐2020‐0941‐0763).

## CONFLICT OF INTEREST

The authors Hao Dong, Zhao‐Liang Li, and Yun‐Yun Zhang were employed by the company Questgenomics, LTD. The author Yan Wang was employed by the company Gnomegen, LLC. The other authors declared no conflict of interest.

## Supporting information


AppendixS1

## Data Availability

The data that support the findings of this study are available from the corresponding author upon reasonable request.
