# Supplementary material for: Assessing early changes in plasma HER2 levels is useful for predicting therapeutic response in advanced breast cancer: A multicenter, prospective, noninterventional clinical study
Source: Cancer Med. 2022 Oct 24;12(5):5323–33. doi: 10.1002/cam4.5352 (PMC10028130; doi:10.1002/cam4.5352)

**Supplementary Methods**

The reference gene was ribonuclease P RNA component H1 (H1RNA) gene (RPPH1). Location: 14q11.2. The determination position is CHR 14:20 343370 on Build GRCh38. It has an 87-bp amplicon that maps within a single exon of the RPPH1 gene.

**Supplemental Tables**

**Supplemental Table S1. Clinicopathological characteristics of patients according to plasma HER2 ratio at baseline**

| Characteristic | n | Plasma HER2 ratio | | | | | |
| --- | --- | --- | --- | --- | --- | --- | --- |
|  |  | HER2 non-amplification ^a^ | |  | HER2 amplification ^a^ | | *P^b^* |
|  |  | n | % |  | n | % |  |
| Total | 85 | 54 | (63.53) |  | 31 | (36.47) |  |
| Age(years) |  |  |  |  |  |  |  |
| ≤45 | 34 | 22 | (64.71) |  | 12 | (35.29) | 0.2800 |
| >45 | 51 | 30 | (58.82) |  | 21 | (41.18) |  |
| Menopausal status |  |  |  |  |  |  |  |
| Premenopausal | 40 | 29 | (72.50) |  | 11 | (27.50) | 0.8580 |
| Postmenopausal | 45 | 25 | (55.56) |  | 20 | (44.44) |  |
| Family history of BC |  |  |  |  |  |  |  |
| No | 65 | 49 | (75.38) |  | 16 | (24.62) | 0.0900 |
| Yes | 20 | 5 | (25.00) |  | 15 | (75.00) |  |
| N stage |  |  |  |  |  |  |  |
| pN1-N2 | 16 | 12 | (75.00) |  | 4 | (25.00) | 0.1130 |
| pN3 | 69 | 42 | (60.87) |  | 27 | (39.13) |  |
| Primary tumor size |  |  |  |  |  |  |  |
| <5cm | 53 | 43 | (81.13) |  | 10 | (18.87) | **0.0030** |
| ≥5cm | 32 | 11 | (34.38) |  | 21 | (65.62) |  |
| Long diameter of the target lesion |  |  |  |  |  |  |  |
| <5cm | 40 | 36 | (90.00) |  | 4 | (10.00) | **0.0001** |
| 5-10cm | 34 | 17 | (50.00) |  | 17 | (50.00) |  |
| ≥10cm | 11 | 1 | (9.09) |  | 10 | (90.01) |  |
| Degree of differentiation |  |  |  |  |  |  |  |
| High and moderate | 45 | 33 | (73.77) |  | 12 | (26.23) | 0.7870 |
| Low | 40 | 21 | (52.50) |  | 19 | (47.50) |  |
| ER |  |  |  |  |  |  |  |
| Negative | 43 | 30 | (69.77) |  | 13 | (30.23) | 0.6510 |
| Positive | 42 | 23 | (54.77) |  | 19 | (45.23) |  |
| PR |  |  |  |  |  |  |  |
| Negative | 45 | 30 | (66.67) |  | 15 | (33.33) | 0.5870 |
| Positive | 40 | 26 | (56.00) |  | 14 | (44.00) |  |
| Ki-67 index |  |  |  |  |  |  |  |
| <14% | 33 | 24 | (72.73) |  | 9 | (27.27) | 0.9660 |
| ≥14% | 52 | 30 | (57.69) |  | 22 | (42.31) |  |
| Liver metastasis |  |  |  |  |  |  |  |
| No | 57 | 38 | (66.67) |  | 19 | (33.33) | 0.5570 |
| Yes | 28 | 16 | (57.14) |  | 12 | (42.86) |  |
| Lung metastasis |  |  |  |  |  |  |  |
| No | 45 | 33 | (73.33) |  | 12 | (26.67) | 0.7080 |
| Yes | 40 | 21 | (52.50) |  | 19 | (47.50) |  |
| Lymph node metastasis |  |  |  |  |  |  |  |
| No | 25 | 20 | (80.00) |  | 5 | (20.00) | 0.3210 |
| Yes | 60 | 34 | (56.67) |  | 26 | (43.33) |  |
| Bone metastasis |  |  |  |  |  |  |  |
| No | 46 | 32 | (69.57) |  | 14 | (30.43) | 0.5380 |
| Yes | 39 | 22 | (56.41) |  | 17 | (43.59) |  |
| Brain metastasis |  |  |  |  |  |  |  |
| No | 73 | 53 | (72.60) |  | 20 | (27.40) | 0.0390 |
| Yes | 12 | 1 | (8.33) |  | 11 | (91.67) |  |
| No. of metastasis sites |  |  |  |  |  |  |  |
| <5 | 40 | 39 | (97.50) |  | 1 | (2.50) | **0.0040** |
| ≥5 | 45 | 15 | (33.33) |  | 30 | (66.67) |  |
| HER2 status by IHC |  |  |  |  |  |  |  |
| 0 or 1+ | 29 | 26 | (89.66) |  | 3 | (10.34) | **0.0001** |
| 2+ | 25 | 11 | (44.00) |  | 14 | (54.00) |  |
| 3+ | 31 | 17 | (54.84) |  | 14 | (45.16) |  |
| HER2 status by FISH |  |  |  |  |  |  |  |
| Non-amplification | 19 | 17 | (89.47) |  | 2 | (10.63) | **0.0080** |
| Amplification | 6 | 2 | (33.33) |  | 4 | (66.67) |  |
| Tumor HER2 status |  |  |  |  |  |  |  |
| Negative | 48 | 44 | (91.67) |  | 4 | (8.33) | **0.0170** |
| Positive | 37 | 10 | (27.03) |  | 27 | (72.97) |  |

^a^ The HER2 amplification status was determined at a cutoff of 1.41.

^b^ *P* values are from the Chi square or Fisher’s exact test and are significant at less than 0.05.

Significant values are shown in bold face.

**Supplemental Table S2. Clinical characteristics of the study population (N=73)**

| **Characteristics** | **N (%)** |
| --- | --- |
| Age (years), median (range) | 52(34-63) |
| Menopausal status |  |
| Premenopausal | 32(43.84%) |
| Postmenopausal | 41(56.16%) |
| Degree of differentiation |  |
| High and moderate | 42(57.53%) |
| Low | 31(42.47%) |
| N stage |  |
| pN1-N2 | 12(16.44%) |
| pN3 | 61(83.56%) |
| Hormone receptor status |  |
| ER and/or PR positive | 41(56.16%) |
| ER and PR negative | 32(43.84%) |
| HER2 status of tumor tissue |  |
| IHC 3+/2+ and FISH+ | 33(42.27%) |
| IHC 0,1+ | 40(57.53%) |
| Phenotype |  |
| Triple-positive | 18(24.66%) |
| HR-, HER2+ | 15(20.55%) |
| HR+, HER2- | 23(31.51%) |
| Triple-negative | 17(23.28%) |
| Ki-67 index |  |
| <14%  ≥14% | 24(32.88%)  52(61.18%) |
| ≥14% | 49(67.12%) |
| No. of metastasis sites | |
| <5 | 34(46.58%) |
| ≥5 | 39(53.42%) |
| Treatment |  |
| Chemotherapy alone | 27(34.25%) |
| Chemotherapy+anti-HER2 | 23(34.25%) |
| Endocrine treatments+anti-HER2 | 7(10.96%) |
| Endocrine treatments+CDK4/6 inhibitors | 14(17.81%) |
| Others | 2(2.73%) |
| Previous lines of therapy in the metastatic setting |  |
| 0 | 7(9.56%) |
| 1 | 31(42.47%) |
| 2 | 25(34.25%) |
| ≥3 | 10(13.72%) |

.

**Supplementary Figure Legends**

Fig. S1 Decrease in plasma HER2 by 15% was set as the threshold for predicting CB in patients with different tissue HER amplification statuses and the exploratory study of the change in the plasma HER2 ratio at 6 weeks from baseline with the best treatment response. (a, c) Value of 15% as the threshold for predicting CB in the tissue HER2 amplification group (N=33, P=0.017) and nonamplification group (N=40, P=0.033) (Fisher’s exact test). (b, d) Predictive value of the 15% threshold for CB in the tissue HER2 ratio amplification group and the nonamplification group according to treatment response. Each data point represents the percent change in the plasma HER2 ratio for a single patient. Horizontal bars represent the median, and error bars indicate the 95% CI. (e, f) Changes in the plasma HER2 ratio at 6 weeks from baseline are shown for patients grouped by treatment response. Each data point represents the percent change in the plasma HER2 ratio for a single patient. Horizontal bars represent the median, and error bars indicate the 95% CI.


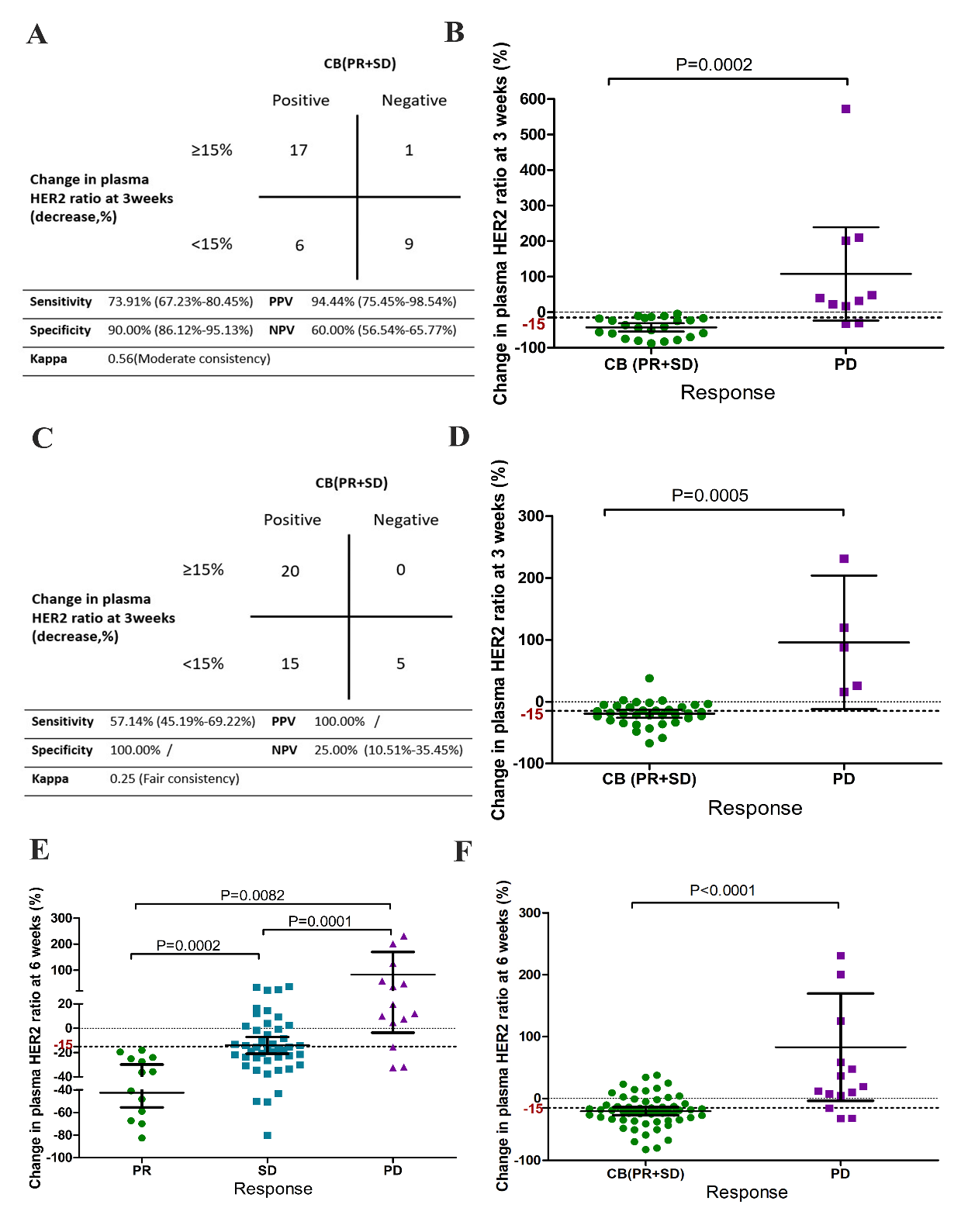


Fig. S2 Increase of 20% as the threshold for predicting PD in patients with different tissue HER amplification statuses. (a, b) Value of 20% as the threshold for predicting PD in the tissue HER2 amplification group (N=33, P<0.0001) and nonamplification group (N=40, P=0.026) (Fisher’s exact test).


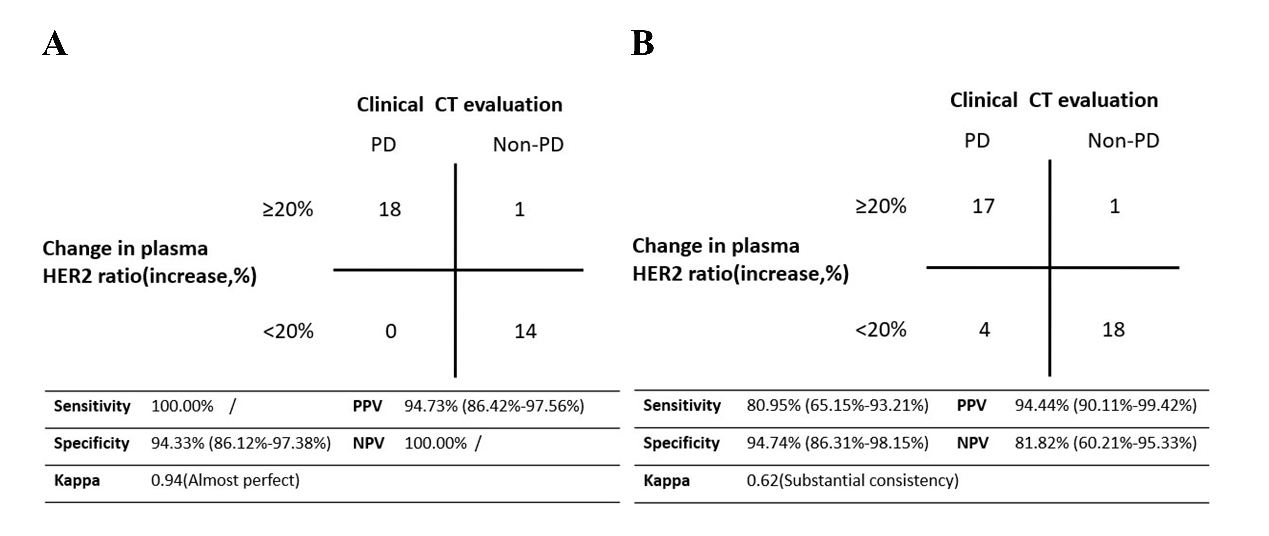

Supplement: Supplementary file 1 — AppendixS1 [file CAM4-12-5323-s001.docx]
